# Supplementary material for: Content-rich biological network constructed by mining PubMed abstracts
Source: BMC Bioinformatics. 2004 Oct 8;5:147. doi: 10.1186/1471-2105-5-147 (PMC528731; doi:10.1186/1471-2105-5-147)
Supplement: Additional File 2 — The original results of the above study (non-essential files are deleted to keep the file size under the limit set by BMC bioinformatics). [file 1471-2105-5-147-S2.bz2 › chilibotAdditionalFile2/dip40/203ID7664335E693/html/ERCC6_CKN1.html]

 


 **ERCC6** and **CKN1** 
  
Found 57 abstracts in PubMed, retrieved 40.  
 

 What does Google say? 
 PDF only 
| .edu only 

---

**Interactive relationship** (e.g. stimulation, inhibition, etc)

**Neutral relationship**- The hydrogen peroxide induced translocation of CSA  [ **CKN1** ]  was also CSB  [ **ERCC6** ]  dependent.  Ref: 11782547 Proc Natl Acad Sci U S A, 2002
- Unlike CSB  [ **ERCC6** ] , CSA  [ **CKN1** ]  does not directly bind Pol II.  Ref: 9372911 Mol Cell Biol, 1997
- Catalase deficient C3H Csb  [ **ERCC6** ]  mutant cell strain is markedly more sensitive to the toxicity of hydrogen peroxide compared to wild type C3H Csa  [ **CKN1** ]  cell strain.  Ref: 9868865 J Radiat Res , 1998
- The translocation of CSA  [ **CKN1** ]  was independent of Xeroderma pigmentosum group C, which is specific to the global genome repair subpathway of nucleotide excision repair and of the core NER factor Xeroderma pigmentosum group A but required the CSB  [ **ERCC6** ]  protein.  Ref: 11782547 Proc Natl Acad Sci U S A, 2002

**Non-interactive relationship** (e.g. studied together, co-existance, homology, etc.)

- The two principle proteins involved in CS, CSA  [ **CKN1** ]  and CSB  [ **ERCC6** ]   **ERCC6** , have been hypothesized to bind RNA polymerase II and link transcription to DNA repair.  Ref: 9372911 Mol Cell Biol, 1997
- Chondroitin sulfate A CSA  [ **CKN1** ] , chondroitin sulfate B CSB  [ **ERCC6** ] , and chondroitin sulfate C CSC are constituents of the basement membranes of many tissues, including the intestine.  Ref: 10100491 Nitric Oxide, 1998
- Cellulose acetate electrophoresis analysis revealed that RPC C2A cells synthesized CSA  [ **CKN1** ]  and CSB  [ **ERCC6** ]  but not CSC.  Ref: 11199713 J Endod, 2000
- Two CS complementation groups, CSA  [ **CKN1** ]  and CSB  [ **ERCC6** ] , have been identified so far.  Ref: 8702468 J Biol Chem, 1996
- The CSA  [ **CKN1** ]  and CSB  [ **ERCC6** ]  genes are implicated in this disease.  Ref: 9278484 Nucleic Acids Res, 1997
- Breakdown of CSA  [ **CKN1** ] , CSB  [ **ERCC6** ] , and CSC was assessed by gel filtration chromatography and compared with untreated controls.  Ref: 10100491 Nitric Oxide, 1998
- Deletion of the gene encoding the Pol II elongation factor TFIIS resulted in enhanced UV sensitivity, but only in the absence of global genome repair dependent on the RAD7 and RAD16 genes, a result seen previously with deletions of RAD26  [ **ERCC6** ]  and RAD28, yeast homologs of the human Cockayne syndrome genes CSB  [ **ERCC6** ]  and CSA  [ **CKN1** ] , respectively.  Ref: 11254132 Mol Gen Genet, 2001
- This phenomenon, referred to as transcription coupled repair requires active transcription, does not require the XPC gene product which is essential for general basal repair reactions, and requires the CSA  [ **CKN1** ]  and CSB  [ **ERCC6** ]  proteins.  Ref: 9065408 J Biol Chem, 1997
- Designated CsA  [ **CKN1** ]  and CsB  [ **ERCC6** ] , their homokaryotic nature was established by polymerase chain reaction amplification and sequence analysis of the allelic variants of three MnP genes.  Ref: 11356573 FEMS Microbiol Lett, 2001
- Finally, there are indications that the genes CSA  [ **CKN1** ]  and CSB  [ **ERCC6** ] , which are implicated in the human hereditary disease Cockayne syndrome, may have a role in transcription.  Ref: 8811173 Annu Rev Biochem, 1996
- The abnormalities in CS are associated with mutations in the CSA  [ **CKN1** ]  or CSB  [ **ERCC6** ]  genes.  Ref: 10739753 Am J Hum Genet, 2000
- Traditionally, CSA  [ **CKN1** ]  and CSB  [ **ERCC6** ]  have been ascribed a function in the transcription coupled repair pathway of nucleotide excision repair that efficiently removes bulky lesions from the transcribed strand of RNA polymerase II transcribed genes.  Ref: 12032859 Oncogene, 2002
- Cockayne syndrome CS is a segmental premature aging syndrome in humans that has two complementation groups, CSA  [ **CKN1** ]  and CSB  [ **ERCC6** ] .  Ref: 12483520 Oncogene, 2002
- Moreover, studies of cells deficient in XPC or in the genes implicated in Cockayne syndrome CSA  [ **CKN1** ]  and CSB  [ **ERCC6** ]  indicated that the drug sensitivity is specifically dependent on the transcription coupled pathway of NER.  Ref: 11479630 Nat Med, 2001
- UV induced ubiquitination of Pol II LS can be restored by introducing cDNA constructs encoding the CSA  [ **CKN1** ]  or CSB  [ **ERCC6** ]  genes, respectively, into CS A or CS B fibroblasts.  Ref: 8876179 Proc Natl Acad Sci U S A, 1996
- Despite this DNA repair defect, individuals with CS of which there are two complementation groups, CSA  [ **CKN1** ]  and CSB  [ **ERCC6** ]  do not demonstrate an elevated incidence of cancer.  Ref: 11713576 Int J Oncol, 2001
- Two complementation groups, CSA  [ **CKN1** ]  and CSB  [ **ERCC6** ] , have been identified.  Ref: 9326587 Proc Natl Acad Sci U S A, 1997
- Defects in nucleotide excision repair NER as defined by the UV sensitivity of xeroderma pigmentosum XP, Cockayne syndrome CS and trichothiodystrophy TTD patients has lead to the identification of most of the genes involved XPA through XPG, CSA  [ **CKN1** ]  and CSB  [ **ERCC6** ] .  Ref: 11104904 Mutat Res, 2001
- Here we demonstrate by microinjection of antibodies against CSB  [ **ERCC6** ]  and CSA  [ **CKN1** ]  gene products into living primary fibroblasts, that both proteins are required for TCR and for recovery of RNA synthesis after UV damage in vivo but not for basal transcription itself.  Ref: 9312053 EMBO J, 1997
- We have tested CSA  [ **CKN1** ]  and CSB  [ **ERCC6** ]  in assays designed to determine their role in transcription coupled repair.  Ref: 9372911 Mol Cell Biol, 1997
- Two of the genes involved, CSA  [ **CKN1** ]  and CSB  [ **ERCC6** ] , are required for transcription coupled repair TCR, a subpathway of nucleotide excision repair that removes certain lesions rapidly and efficiently from the transcribed strand of active genes.  Ref: 10564257 Mol Biol Cell, 1999
- However, inhibition was lower with CSA  [ **CKN1** ]  and CSB  [ **ERCC6** ]  than that observed with HEP and HSA.  Ref: 10474198 FEMS Microbiol Lett, 1999
- Alternatively, these findings may reflect a role played by the CSA  [ **CKN1** ]  and CSB  [ **ERCC6** ]  gene products in transcription.  Ref: 8876179 Proc Natl Acad Sci U S A, 1996
- Two genes are involved CSA  [ **CKN1** ]  and CSB  [ **ERCC6** ] .  Ref: 12560492 Nucleic Acids Res, 2003
- Our analysis will assist the identification of mutations in the recently cloned CSA  [ **CKN1** ]  and CSB  [ **ERCC6** ]  genes and the study of structure function relationships.  Ref: 8834235 Hum Genet, 1996
- 10 ng ml of TGF beta increased the production of CSA  [ **CKN1** ]  and CSB  [ **ERCC6** ]  in the cell ECM fraction.  Ref: 11199713 J Endod, 2000
- Lack of inhibition by added CSA  [ **CKN1** ] , CSB  [ **ERCC6** ] , and CSC in contrast to CSE strongly suggests a direct role of di O sulfated N acetylgalactosamine GAG structures in the inhibition of intrinsic pathway protease.  Ref: 7592813 J Biol Chem, 1995
- In this study, we show that two chondroitin 4 sulfate forms, CSA  [ **CKN1** ]  and CSB  [ **ERCC6** ] , can activate distinct peripheral blood mononuclear cell types.  Ref: 9639520 Blood, 1998
- Gel filtration of repair and transcription competent whole cell extracts provided evidence that CSB  [ **ERCC6** ]  and CSA  [ **CKN1** ]  are part of large complexes of different sizes.  Ref: 9312053 EMBO J, 1997
- Heparin HEP, heparan sulfate HSA, chondroitin sulfate A CSA  [ **CKN1** ]  and chondroitin sulfate B CSB  [ **ERCC6** ]  inhibited adherence and internalization of S. uberis in a dose dependent manner.  Ref: 10474198 FEMS Microbiol Lett, 1999
- The disease is caused by the disruption of CSA  [ **CKN1** ] , CSB  [ **ERCC6** ] , or some types of xeroderma pigmentosum genes.  Ref: 12665480 FASEB J, 2003
- ... survival, repair and recovery from transcription inhibition critically depend on CSA  [ **CKN1** ] , CSB  [ **ERCC6** ]  and UVS, the factors specific for transcription coupled NER.  Ref: 12531012 DNA Repair Amst , 2002
- Despite this DNA repair defect, individuals with CS group A CSA  [ **CKN1** ]  or group B CSB  [ **ERCC6** ]  do not exhibit an increased spontaneous or UV induced cancer rate.  Ref: 11238917 Mol Cell Biol, 2001
- These findings indicate that CSA  [ **CKN1** ]  and CSB  [ **ERCC6** ]  are major sulfated GAGs synthesized by RPC C2A cells.  Ref: 11199713 J Endod, 2000
- In addition to interacting with XPA, immunoprecipitation experiments demonstrated that a fraction of XAB2 is able to interact with the transcription coupled repair specific proteins CSA  [ **CKN1** ]  and CSB  [ **ERCC6** ]  as well as RNA polymerase II.  Ref: 10944529 J Biol Chem, 2000
- The chondroitin sulfates CSA  [ **CKN1** ] , CSB  [ **ERCC6** ] , CSC had far less impact upon APTT with the FXa FXDP system.  Ref: 12430983 Can J Physiol Pharmacol, 2002
- The UV induced degradation rates of RNAP II LS were not significantly altered when comparing CSA  [ **CKN1** ]  or CSB  [ **ERCC6** ]  to repair competent control cells.  Ref: 11516929 Mutat Res, 2001
- Dental pulps contain sulfated glycosaminoglycans GAGs, such as chondroitin 4 sulfate CSA  [ **CKN1** ]  4CS, dermatan sulfate CSB  [ **ERCC6** ]  DS, and chondroitin 6 sulfate CSC 6CS.  Ref: 11199713 J Endod, 2000
- Whereas CSA  [ **CKN1** ]  activates monocytes to secrete monokines, CSB  [ **ERCC6** ]  activates B cells to proliferate.  Ref: 9639520 Blood, 1998
- Measurement of 35S sulfate incorporation showed a significant increase in the amount of GAGs by TGF beta, 1.3 fold CSA  [ **CKN1** ] , and 1.2 fold CSB  [ **ERCC6** ]  in the cell ECM fraction.  Ref: 11199713 J Endod, 2000
- The abnormalities in CS are caused by mutated CSA  [ **CKN1** ]  or CSB  [ **ERCC6** ]  genes.  Ref: 11443545 Am J Hum Genet, 2001
- The NO donor SNAP 2 mM, pH 4.0 or PN 5 mM, pH 7.4 was incubated for at least 1 week at 37 degrees C with CSA  [ **CKN1** ] , CSB  [ **ERCC6** ] , or CSC.  Ref: 10100491 Nitric Oxide, 1998
- Comparison of the activities of CSA  [ **CKN1** ]  and CSB  [ **ERCC6** ]  oligo and polysaccharides with a similar sulfation pattern and content achieved from partial desulfation demonstrated that glucuronic acid rather than iduronic acid residues are important for IRBC binding.  Ref: 11956186 J Biol Chem, 2002
- Two genes have been shown to be involved CSA  [ **CKN1** ]  and CSB  [ **ERCC6** ] .  Ref: 9565609 J Biol Chem, 1998
